# Age-related changes in gait, balance, and strength parameters: A cross-sectional study

**DOI:** 10.1371/journal.pone.0310764

**Published:** 2024-10-23

**Authors:** Asghar Rezaei, Sandesh G. Bhat, Chih-Hsiu Cheng, Robert J. Pignolo, Lichun Lu, Kenton R. Kaufman

**Affiliations:** 1 Department of Orthopedic Surgery, Division of Orthopedic Research, Mayo Clinic, Rochester, MN, United States of America; 2 Department of Physiology and Biomedical Engineering, Mayo Clinic, Rochester, MN, United States of America; 3 School of Physical Therapy and Graduate Institute of Rehabilitation Science, College of Medicine, Chang Gung University, Linkou, Taoyuan, Taiwan; 4 Bone and Joint Research Center, Chang Gung Memorial Hospital, Linkou, Taoyuan, Taiwan; 5 Department of Medicine, Divisions of Geriatric Medicine and Gerontology, Endocrinology and Hospital Internal Medicine, and the Robert and Arlene Kogod Center on Aging, Mayo Clinic, Rochester, Minnesota, United States of America; University of Illinois Urbana-Champaign, UNITED STATES OF AMERICA

## Abstract

**Background:**

Longevity is increasing worldwide due to improvements in healthcare and living standards. Aging is often associated with disability and multiple health concerns. To address these challenges, effective interventions are essential. This study investigated potential age-related declines in gait, balance, and strength. We also sought to assess any relationships between these three parameters and explore potential differences between women and men.

**Methods:**

Healthy individuals over 50 years of age were recruited for this cross-sectional study. Upper extremity (grip) strength and lower extremity (knee) strength of the dominant side were measured. Static balance was performed on the force plate in different situations each for 30 seconds: bilateral stance with eyes open, bilateral stance with eyes closed, as well as dominant leg and non-dominant leg unilateral stance with eyes open. Gait was measured during level walking using an optical motion capture system. Additionally, the dynamic stability margin (DSM) was calculated for the level walking trials.

**Results:**

The study results indicated that gait parameters were not significantly affected by age (p≥0.12), while knee and grip strength, along with several balance parameters, showed a significant decline with age. All individuals were able to maintain their bipedal balance, but their center of pressure movement increased significantly by age (p≤0.028). Z-scores were calculated to compare significant age parameters. Unipedal stance time was found to be the most affected by age compared to other contributing factors (p≤0.001). The duration of unipedal balance showed the most significant change per decade (non-dominant: -0.62 SDs; dominant: -0.53 SDs), while strength measures exhibited the lowest amount of change per decade (grip strength: -0.34 SDs; knee strength: -0.26 SDs). Sex differences were observed exclusively in strength parameters, with no discernible impact on the decline in balance parameters.

**Conclusions:**

These findings suggest that the duration of unipedal stance can serve as a reliable and gender-independent measure of neuromuscular aging for both elderly male and female subjects.

## 1. Introduction

Longevity is increasing worldwide as a result of improvements in healthcare and living standards [1]. Aging causes a decline in mental and physical capacity [2]. Gradual loss of physical activities is a characteristic of aging [3]. Sarcopenia is one of the most devastating impacts of aging, resulting in the loss of muscle mass, strength, and function [4]. While aging is not a disease, it can be associated with disability and multimorbidity [5]. Adequate muscle strength, efficient gait, and good balance, which decline with age, are crucial contributors to independence and well-being. Investigating how aging deteriorates the parameters related to balance and strength is crucial for both patients and clinicians.

Gait is an important aspect of daily activity that affects quality of life in the elderly. Gait is significantly affected in older adults [2]. Gait disturbances may manifest in old age due to various factors such as sensory deficits (e.g., visual impairment). Gait speed has been shown to decline with age, especially after age 65 [6]. Slow walking is associated with functional decline and poorer physical health [7]. Aranda-Garcia et. al. showed that knee strength was the best predictor of gait speed in older rural women, while recommending future examination of gait speed in older men [8]. Hence, gait speed is an important outcome measure when studying effects of aging.

Balance refers to the ability to maintain equilibrium or postural control [9]. Balance is a complex activity that integrates information from vision, the vestibular system, and the somatosensory system to sense positions, velocities, and accelerations. This enables individuals to maintain posture and respond to voluntary movements and gait disturbances [10]. Static balance is necessary for maintaining postural control during standing and some physical activities and dynamic balance is crucial to control the body’s center of mass (CoM) during mobility [9]. Park et. al. studied gait and dynamic balance in adults between ages 21 to 89 years but did not quantify static balance in the cohort [11]. El Haber et. al. performed both dynamic and static balance tests, but did not consider unipedal balance [12]. Balance impairments can lead to falls, both while stationary and while moving. Elderly individuals are at an increased risk of bone fractures with serious consequences due to osteoporosis [13,14], making falls a severe health risk.

The rate of decline in muscle mass is up to 8% per decade after the age of 30, and this rate increases after the age of 60 [15]. Goodpastor et. al. concluded that along with loss of muscle mass, decline in muscle quality may also affect strength with aging [16]. They did not study balance or gait measures in their study cohort. Elderly individuals experience a significant decline in muscle mass and strength over time, reaching a disability threshold [17]. Therefore, a reduction in muscle strength is closely associated with the loss of independence and a diminished quality of life.

Although gait, muscle strength, and balance decline with age, which of these parameters deteriorates faster and at what rate? Answers to these questions can help healthcare professionals design targeted interventions that more effectively slow down these declines by offering maintenance and training programs. Despite previous studies investigating multiple age-related factors for gait, balance, and strength measures, a hierarchy for the measures has not been discussed, and the rate of declines have not been compared in healthy elderly. Studies have also focused only on effective interventions, informed by monitoring the effects of aging, that are necessary to delay or reverse the onset of changes associated with aging. In this cross-sectional study, our aim was to investigate how aging affects gait, balance, and strength in a healthy, independent adult cohort and compare the rates of age-related decline associated with these parameters. Additionally, we sought to assess any relationships between these three parameters, explore potential differences between women and men, and establish a hierarchy among the studied measures. In addition, multiple balance tests were performed, and upper and lower extremity strength parameters were measured and compared. Gait analysis and multiple balance assessments were conducted on a healthy adult cohort using a motion capture system and force plates.

## 2. Methods

### 2.1. Participants

In this cross-sectional study, a convenience sampling technique was used to select individuals located in Rochester, MN. Human subjects provided written consent using IRB 20–013160. Several modes of recruitment were utilized, such as posting a flyer on the Mayo Clinic website, reaching out to individuals from previous studies and the Rochester-area Older Adult Registry (ROAR). ROAR is a Mayo Clinic community-based, longitudinal primary care population of adults ≥ 65 years of age that collects information to address scientific questions on determinants of healthy aging. Recruitment commenced on March 10, 2022, and data collection continued until March 24, 2023. Individuals over the age of 50 were recruited after securing institutional review board approval. To achieve a uniform distribution of ages, the number of subjects aged 50 to 64 were equal to the number of those aged 65 and older for each sex. Individuals with a BMI of greater than 35, difficulty performing common activities of daily living, spinal pathologies, neuromuscular disorders, used assistive walking devices, or FRAIL score of 3 or greater were excluded [18]. Demographic data such as age, height, weight, BMI, trochanteric height, activity levels (using the International Physical Activity Questionnaire (IPAQ)–Short Form) [19], and limb dominance were also collected and analyzed. The physical activity measures included the self-reported time spent sitting (hours/day) as well as the assigned category of physical activity (high, moderate, or low) based on their reported activity time in combination with the weighting factors for different activities. IPAQ guidelines were used to calculate the continuous score of the metabolic equivalents (MET) per time. The combined Total physical activity (MET-minute/week) was computed as the sum of the scores of the three activities [20].

Data collections was performed at the Mayo Clinic Motion Analysis Laboratory. Marker placements were performed by trained physiotherapists. A total of 36 spherical reflective markers were used; markers were attached to feet, shanks, thighs, pelvis, trunk, and head to represent the entire body. All bony landmarks were identified following palpation guidelines. The female subjects participating in the study wore custom-made open back gowns, which facilitated marker placement. Marker trajectory data were captured using a 14-camera Real-Time Motion Analysis system (Raptor 12HS, Motion Analysis, Rohnert Park, CA). The order of the tests was walking, balance, grip strength, and knee strength. Walking and balance tests were performed with all the markers attached to the body at the subject’s own pace. Then, markers were removed, and individuals were asked to perform strength measurements, with a rest time of five to ten minutes between each strength test. Mock trials were performed in advance of data collection. Additionally, all the equipment was calibrated according to manufacturers’ instructions before any use.

### 2.2. Strength measurements

Isometric upper extremity (grip) strength was assessed with a custom-made device (NK Biotechnical Corporation, Minneapolis, MN) with the subject seated upright in a hardback chair with elbow at 90° flexion, wrist in neutral position, and feet flat on the floor [21,22]. Isometric lower extremity (knee) extension strength was measured using the Humac Norm system (CSMi Medical Solutions, Stoughton, MA) according to a previous study [22,23]. The subject was positioned with both hip and knee were at 90° flexion and instructed to extend their knee as quickly as possible. Both tests were carried out on the dominant side. Three trials were conducted for each strength test with a time interval of at least 30 seconds between each trial, and the maximum value was selected for the analysis.

### 2.3. Standing balance

As previously explained [22], static balance was performed on the force plate in different situations each for 30 seconds: bilateral stance with eyes open, bilateral stance with eyes closed, as well as dominant leg and non-dominant leg unilateral stance with eyes open. Participants were guided by a physical therapist during each test. For all balance testings, participants were instructed to look straight ahead with arms on their sides. For bilateral balance tests, individuals were asked to stand still with both feet on two force plates (Kistler Instrument Corp., Amherst, NY), while eyes were open or closed. For one-leg standing trial, individuals were instructed to step on one leg with eyes open. The position of the non-load bearing leg and arms was selected by the subject to maintain balance. The aggregate center of pressure (CoP) data from force plates were calculated at 600 Hz. The postural sway in the subjects during both bipedal and unipedal standing balance was quantified using the root mean square of the center of pressure (RMS(CoP)). The order of the balance tests was the non-dominant side first. The amount of movement in the center of pressure (Path(CoP)) was integrated to quantify the distance the CoP moved during the balance trials. The duration of unipedal balance was calculated as the maximum duration the subject was able to keep balance, which was used as an outcome measure. The ratio of the (RMS(CoP)) with eyes closed and eyes open, which is known as the Romberg ratio, was also calculated [24].

### 2.4. Gait parameters

Gait parameter/s were measured during level walking. Participants were asked to walk back and forth on an eight-meter walkway at a self-paced, preferred speed. Gait parameters were calculated from the marker trajectories that were obtained by the motion capture system at 120 Hz. Three walking trials were collected for each subject for data analysis. Gait outcome measures are summarized in Table 1 [22]. Force plate data were collected along with gait parameters during walking trials. The tools used in the current study are commonly used and have been validated extensively [25–34].

**Table 1:** Normalization techniques for the outcome measures according to Hof [35].

Variable	Definition	Unit	Normalization
**Strength measures**			
Dominant grip strength	Maximum dominant side grip strength value out of three trials	Kg	(mass/100)^−1^
Dominant knee strength	Maximum dominant side knee strength value out of three trials	Nm	(mass.g.leg/100)^−1^
**Bipedal standing balance**			
RMS(CoP)_EO_	Average standing postural sway with eyes open on both legs	m	(leg/100)^−1^
RMS(CoP)_EC_	Average standing postural sway with eyes closed on both legs	m	(leg/100)^−1^
RMS(CoP)_EC/EO_	Romberg ratio of the standing postural sway on both legs	m	(leg/100)^−1^
Path(CoP)_EC_	Amount of movement in the CoP with eyes open on both legs	m	(leg/100)^−1^
Path(CoP)_EO_	Amount of movement in the CoP with eyes closed on both legs	m	(leg/100)^−1^
Path(CoP)_EC/EO_	Romberg ratio of the amount of movement in the CoP when standing on both legs	m	(leg/100)^−1^
**Unipedal standing balance**			
RMS(CoP)_Dominant_	Average standing postural sway with eyes open on the dominant leg	m	(leg/100)^−1^
RMS(CoP)_NonDominant_	Average standing postural sway with eyes open on the non-dominant leg	m	(leg/100)^−1^
Duration(balance) _Dominant_	Duration of balance on the dominant leg	s	${\left(\sqrt{leg/g}\right)}^{-1}$
Duration(balance) _NonDominant_	Duration of balance on the non-dominant leg	s	${\left(\sqrt{leg/g}\right)}^{-1}$
**Gait parameters**			
Gait speed	Distance traveled per time unit	m/s	${\left(leg.\sqrt{leg/g}\right)}^{-1}$
Cadence	Number of steps per time unit	1/s	$\sqrt{leg/g}$
Stride length	Sagittal distance between successive heel strikes of same foot	m	(leg/100)^−1^
Step width	Lateral distance between successive heel strikes of two feet	m	(leg/100)^−1^
Gait stability ratio	Cadence divided by gait speed	1/m	leg/100
Single support	One foot in touch with the ground	%	-
Double support	Both feet in touch with the ground	%	-
**Dynamic gait balance**			
StepLength_right_	Distance between two consecutive steps on the right side	m	(height)^−1^
StepLength_left_	Distance between two consecutive steps on the left side	m	(height)^−1^
DSM_right_	Shortest distance from the xCoM to the BoS during the gait cycle for the right leg	m	(height)^−1^
DSM_left_	Shortest distance from the xCoM to the BoS during the gait cycle for the left leg	m	(height)^−1^

### 2.5. Dynamic balance

The dynamic stability margin (DSM) was calculated for the level walking trials using the procedure described in Simon et. al.’s article [36]. The step length, Base of Support (BoS), and the extrapolated center of mass (xCoM) [37,38] were determined based on the coordinates of reflective markers. To calculate the xCoM, it was necessary to estimate both the position and velocity of the overall body’s Center of Mass. This involved employing a 13-segment rigid body model to compute the weighted sum of the entire body’s CoM [39]. Additionally, the boundaries of the Base of Support were established by utilizing four markers positioned on each foot. The DSM was calculated using the below formula:

DSM=xCoM−BoS

The calculated outcome measures were normalized using appropriate body parameters, as prescribed in literature [35], for each subject to account for different body dimensions (Table 1).

### 2.6. Statistical analysis

The demographic variables (height, weight, BMI, trochanteric height, and activity level) were analyzed using an ANOVA test between the two groups (below 65 and above 65). Linear regression was used to analyze the activity level with respect to age. Linear regression was also used to analyze the relationship between the outcome variables (listed in Table 1) and main independent variables (Age and Sex). The interaction of the independent variables was included in the linear regression only if the term was significant. An ANCOVA analysis was used to adjust the amount of center of pressure movement for the unipedal standing duration. A z-score was calculated for the outcome measures that were found to be significantly related to Age. These z-scores were then compared to identify the hierarchy of outcome measures related to aging. All statistical analyses were performed in R [40].

### 2.7. Sample size calculation

Since the primary analysis performed is a linear regression analysis, sample size calculation techniques described by Cohen et. al. were utilized [41]. The R^2^ values for gait speed, and balance were obtained from the literature as 0.23 [6], and 0.271 [11]. The sample size (N) was calculated as $N=\frac{L}{f^{2}}+k+1$, where L is the non-centrality parameter selected from the appendix table E.2 [41], f^2^ is the, and k is the number of predictor. The values of L, f^2^, and k were calculated to be 9.64, 0.298, and 2 for gait speed, resulting in a sample size of 36. The values of L, f^2^, and k were calculated to be 9.64, 0.3717, and 2 for gait speed, resulting in a sample size of 29. Rounding up the highest calculated sample size, we get a target sample size of 40.

## 3. Results

A total of 40 subjects (Table 2) signed an informed consent form before being enrolled in the clinical study. The subjects below 65 years of age and above 65 years of age were statistically similar in terms of height, weight, BMI, trochanteric height, and activity levels. The activity level in the recruited subjects was not related to the subject’s age (R^2^ = 0.02, p = 0.395). Hence, activity level was not considered as a factor in the forthcoming linear regression analyses.

**Table 2:** Demographic data of the subjects.

	Below 65	Above 65	p-value
Participants (Male/Female)	20 (10/10)	20 (10/10)	-
Age (years)	56 (4)	74 (5)	-
Height (cm)	171.2 (10.9)	168.6 (8.2)	0.397
Trochanteric height (cm)	91.8 (5.1)	90 (6)	0.327
Weight (kg)	78.3 (13.6)	77.8 (12.7)	0.911
BMI (kg/m^2^)	26.6 (3.3)	27.4 (4.1)	0.516
Activity Level (IPAQ MET minutes per week)	4180 (2878)	3269 (3083)	0.34
Dominant side (Right/Left)	(17/3)	(20/0)	-

BMI: Body Mass Index; IPAQ: International Physical Activity Questionnaire; MET: Metabolic Equivalent of Task.

### 3.1. Strength measurements

**Fig 1:**
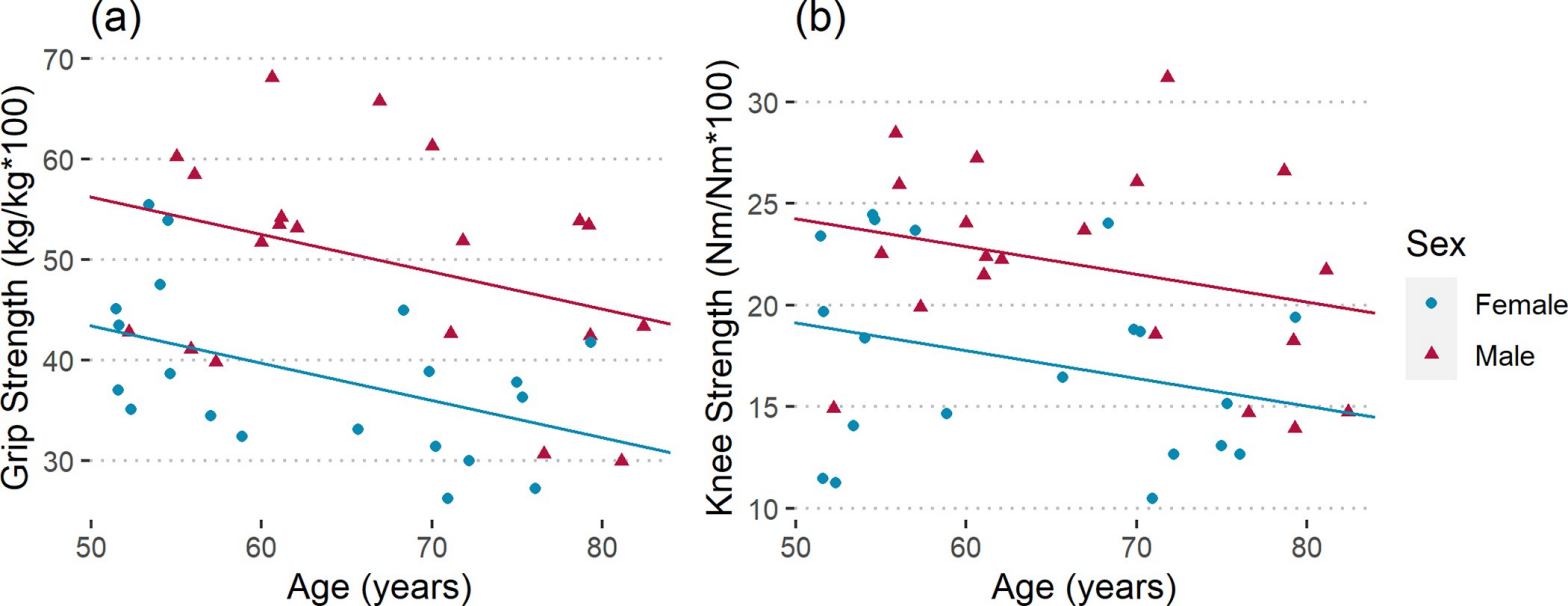
Normalized (a) dominant grip strength (R^2^ = 0.39, p < 0.001) and (b) dominant knee strength (R^2^ = 0.25, p = 0.005) for male and female subjects.

There was a significant relationship between the strength measures and Age and Sex (dominant grip strength: R^2^ = 0.39, p < 0.001; dominant knee strength: R^2^ = 0.25, p = 0.005). The dominant grip strength declined at a rate of 3.7% (kg/kg) per decade for both sexes (Fig 1A). Men had 30% higher grip strength than women. Knee strength declined at a rate of 1.4% (Nm/Nm) per decade (Fig 1B). Men had 27% higher knee strength than women. Hence, dominant grip and knee strength are a good indicator of age-related musculoskeletal changes in both sexes.

### 3.2. Standing balance

#### Bipedal

Average standing postural sway (RMS(CoP)) was not related to Age and Sex (eyes open: R^2^ = 0.04, p = 0.47; eyes closed: R^2^ = 0.17, p = 0.09). The Romberg ratio (RMS(CoP)_EC/EO_) also was not related to either Age or Sex (R^2^ = 0.02, p = 0.71). Hence average standing postural sway is not a valid measure of aging related changes in either sex.

**Fig 2:**
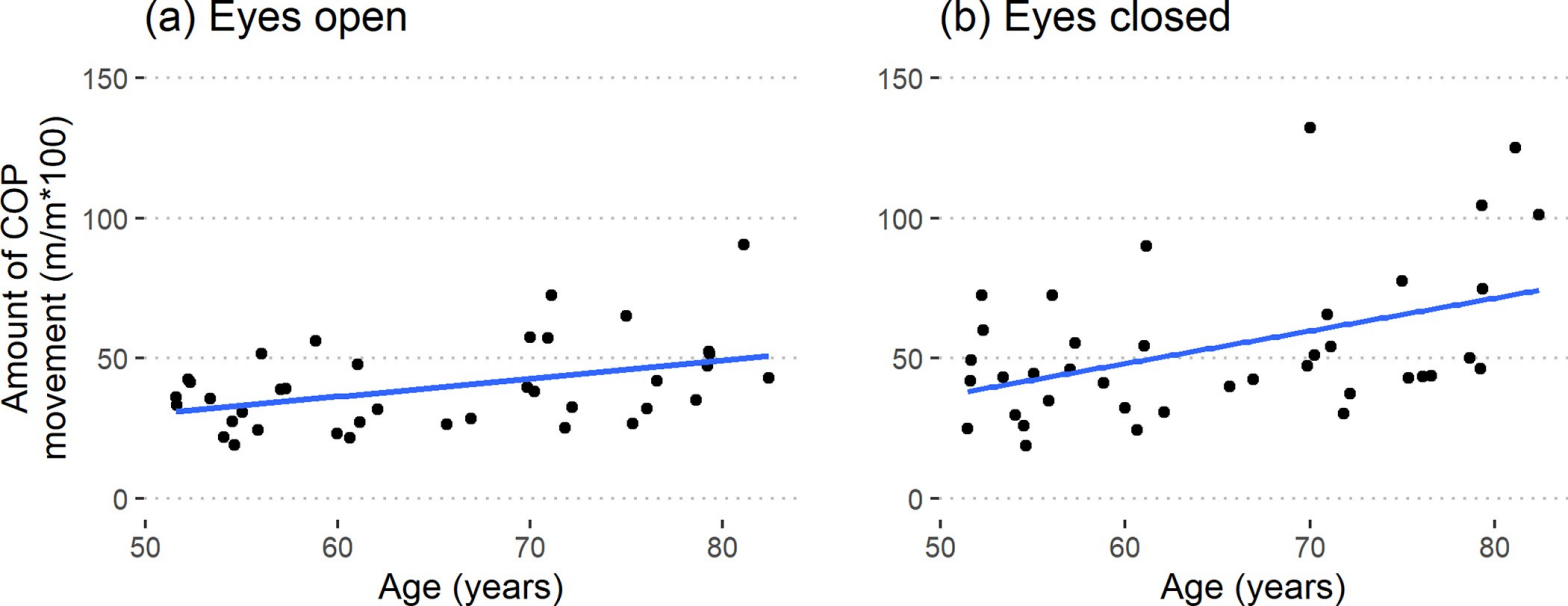
The amount of movement in the center of pressure for (a) Eyes open (R^2^ = 0.18, p = 0.028) and (b) eyes closed (R^2^ = 0.25, p = 0.005) condition.

The amount of movement in the CoP increased at a rate of 6.3% (m/m) per decade for the eyes open condition (R^2^ = 0.18, p = 0.028) in both the sexes (Fig 2A). Whereas for the eyes closed condition, the increase was 10.4% (m/m) per decade (R^2^ = 0.25, p = 0.005) in both the sexes (Fig 2B). The Romberg ratio (Path(CoP)_EC/EO_) was not related to Age or Sex (R^2^ = 0.06, p = 0.33). Hence, the older subjects moved more while standing on both their legs compared to the younger subjects for both conditions.

#### Unipedal

**Fig 3:**
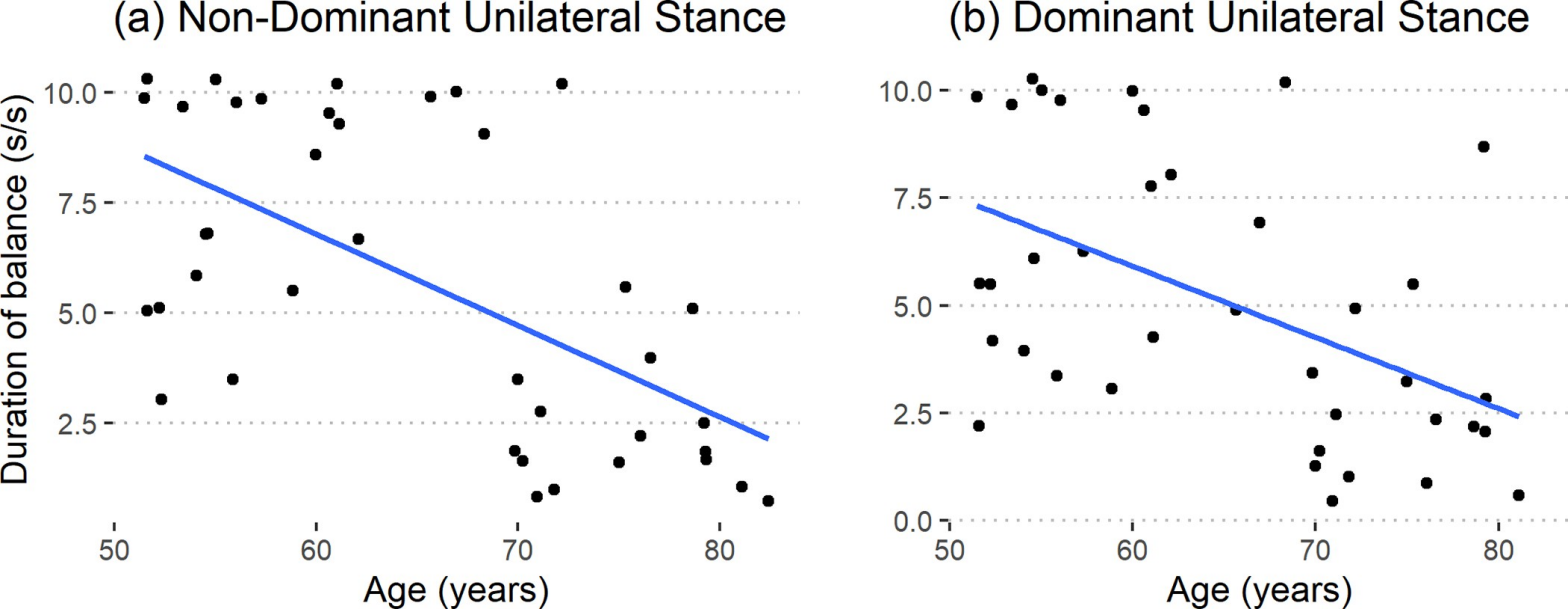
Unipedal standing duration for the (a) non-dominant (R^2^ = 0.38, p < 0.001) and (b) dominant sides (R^2^ = 0.27, p = 0.004).

Average postural sway when balancing on one leg was not related to age or sex (non-dominant: R^2^ = 0.09, p = 0.206; dominant: R^2^ = 0.13, p = 0.074). Hence, the average postural sway is not a good predictor of neuromuscular aging. The amount of movement of the center of pressure, while balancing on one leg, was only dependent on the unipedal standing duration (p < 0.001) and had no relation with Age or Sex (p > 0.1). Unipedal standing duration, when normalized, declined at the rate of 2.2 (s/s) per decade in the non-dominant side (R^2^ = 0.38, p < 0.001) and at the rate of 1.7 (s/s) per decade in the dominant side (R^2^ = 0.27, p = 0.004) for both the sexes (Fig 3). Hence, the duration the subjects could balance on one leg deteriorated with age.

### 3.3. Gait analysis

None of the gait parameters were related to Age (Table 3). The percent double support was different for both men and women (p = 0.002) but was not related to Age. Hence, gait parameters were not a good indicator of neuromuscular aging.

**Table 3:** Linear regression results for gait parameters.

Variable	R^2^	p-value
Gait speed	0.05	0.4
Cadence	0.08	0.19
Stride length	0.03	0.52
Step width	0.11	0.12
Gait stability ratio	0.03	0.6
Single support	0	0.85
Double support	0.22	0.009

### 3.4. Dynamic balance

The step lengths were similar for all ages and sexes on the right and left sides (right: R^2^ = 0, p = 0.95; left: R^2^ = 0.01, p = 0.76). The measure of dynamic balance during gait (DSM) was also similar for all ages and sexes on the right and left sides (right: R^2^ = 0.05, p = 0.38; left: R^2^ = 0.09, p = 0.18). Hence, the ability to balance during gait did not change considerably with age in both male and female subjects.

### 3.5. Z-score comparison

Z-scores were calculated for dominant grip strength, dominant knee strength, amount of movement of the center of pressure during bipedal standing with and without eyes closed, and the duration of unipedal balance on both legs. The duration of unipedal balance showed the most change per decade (non-dominant: -0.62 standard deviations; dominant: -0.53 standard deviations) followed by the amount of movement of the center of pressure during bipedal standing (eyes open: 0.41 standard deviations; eyes closed: 0.39 standard deviations). The strength measures showed the lowest amount of change per decade (dominant grip strength: -0.34 standard deviations; dominant knee strength: -0.26 standard deviations).

## 4. Discussion

The study collected objective data from gait analysis, balance tests, and upper and lower extremity strength measurements to investigate the effect of aging on gait, balance, and strength in a healthy, independent adult cohort and compare the rates of age-related decline associated with these parameters. Also, we sought to establish a hierarchy among the studied measures. While gait parameters did not change with age, we found several balance and strength metrics that showed significant age-related declines. Among these factors, unipedal balance time on the non-dominant side was most affected by age, while knee strength was affected the least. Despite the ability to maintain balance during bipedal stance, the CoP moved significantly more with increasing age.

Measuring age-related determinants is crucial in managing elderly patients [42]. Assessment of these factors is typically complex, necessitating specialized tools and protocols that need to be executed by expert clinicians to ensure repeatability and reproducibility. Differences in definitions, measurement tools, and protocols for assessing aging factors make comparisons across multiple studies challenging. Importantly, the current study identified the duration a person can maintain balance on the non-dominant leg as the factor with the highest rate of decline. This finding is significant because this measurement does not require specialized expertise, advanced tools, or techniques for measurement and interpretation. It can be easily performed, even by individuals themselves.

Elderly subjects can gain balance and strength through laboratory-based training programs and maintain those gains with maintenance programs [43]. The results of the current study can help optimize these training and maintenance programs to improve balance and strength in the elderly population, thereby postponing or avoiding disability.

Unipedal stance time is a valid measure of frailty, independence, and fall status [44,45] and proves to be a useful tool in identifying patients with peripheral neuropathy [46]. Despite its significance, decline in unipedal stance time has not been adequately studied in the context of aging. The importance of balance, especially in unipedal stance, arises from the fact that it requires multiple sensory inputs and neuromuscular control, in addition to adequate muscle strength. This is why balance on one leg, as demonstrated in our study, undergoes the fastest decline in our healthy cohort, reflecting age-related declines in muscle strength similar to prior studies [15], and in the rapid coordination and integration of data by the central nervous system. To the best of our knowledge, such a comparison is the first of its kind within the elderly population.

While all the subjects were easily able to maintain their balance during bilateral stance tests, our results showed that their CoP movements increased significantly with age. Our results were in accordance with prior studies, showing that the bipedal balance deteriorated with age [12,47–49] While aging affects both muscular and neurological aspects, standing on both limbs for only 30 seconds does not require a large amount of muscle strength. This can mean that increased CoP movement due to aging may suggest a greater decline in the neuromuscular sensory system compared to strength. Hernandez et al.’s study showed that although velocity of movement was lower when compared to younger subjects, the older subjects performed frequent movements to maintain their balance [49]. This signifies how aging affects our ability to maintain balance.

Muscle strength serves as an additional indicator of muscle quality and a predictor of various health concerns, such as disability and mortality [50]. Unlike level walking or balance tests, maximal muscle strength evaluates the greatest capacity of the muscle, which declines with age. The grip strength test, a simple and reliable measurement [51], has been recognized as a powerful predictor of disability, mortality, and morbidity [52]. The current study observed a significant decline in grip strength, which decreased at a faster rate than knee strength. This trend aligns with findings from a longitudinal study, where grip strength was reported to decline more rapidly than hip or knee strength [53]. Hence, grip strength serves as a better predictor of musculoskeletal aging than other strength measures.

Sex differences were observed in knee and grip strength parameters, even when the strength data were normalized by the weight and size of individuals. However, there were no sex-specific age-related declines in strength parameters, indicating that all individuals experienced declines in upper and lower extremity strength at a similar rate. This was similar to Haynes et al.’s study, which indicated that there was no sex difference in isometric knee extension and flexion strength in subjects above 60 years of age [54]. No sex differences were identified in the gait and balance parameters studied in the current article, suggesting that both male and female subjects were equally affected by age similar to prior studies [55].

The primary limitation of this study lies in its cross-sectional design, which poses challenges in accounting for potential confounding variables. Although participants were randomly selected from Mayo Clinic patients in Rochester MN, the cohort’s representativeness for the broader population may be limited. Another limitation could be the order of evaluation, which might have constituted a risk of bias as the participants performed all the tasks in one visit. To mitigate bias, evaluations started with walking followed by balance tests. Then, reflective markers were removed from the subject, providing enough time to rest before strength analysis. Grip strength was assessed before knee strength, providing several minutes of rest while preparing for the knee test. This strategy minimized evaluation bias.

## 5. Conclusions

This study underscores the significance of the unipedal balance test in monitoring elderly subjects in the community, regardless of sex. The duration an individual, whether male or female, can maintain balance on one leg emerges as the most reliable determinant of aging, surpassing strength, gait, and other balance parameters.

## Supporting information

S1 File (XLSX)

S1 Dataset (XLSX)
