# Correction: Age-related changes in gait, balance, and strength parameters: A cross-sectional study

**DOI:** 10.1371/journal.pone.0344583

**Published:** 2026-03-09

**Authors:** Asghar Rezaei, Sandesh G. Bhat, Chih-Hsiu Cheng, Robert J. Pignolo, Lichun Lu, Kenton R. Kaufman

The image for Fig 3 is incorrect. Please see the correct Fig 3 here.

In Table 1, the data in the columns 3 and 4 for the variable “RMS(CoP)_EC/EO_” and “Path(CoP)_EC/EO_” are incorrect. Please see the correct Table 1 here.

In the Bipedal subsection of the Results, there is an error in the third sentence of the second paragraph. The correct sentence is: The Romberg ratio (Path (CoP)EC/EO) was not related to Age or Sex (R^2^ = 0.09, p = 0.21).

In the Unipedal subsection of the Results, there is an error in the fourth sentence of the paragraph. The correct sentence is: Unipedal standing duration, when normalized, declined at the rate of 21 (s/s) per decade in the non-dominant side (R2 = 0.38, p < 0.001) and at the rate of 17 (s/s) per decade in the dominant side (R2 = 0.27, p = 0.004) for both the sexes.

**Table 1:** Scaling techniques for the outcome measures according to Hof [35].

Variable	Definition	Unit	Scaling Parameters
**Strength measures**			
Dominant grip strength	Maximum dominant side grip strength value out of three trials	Kg	$(\text{M}{)}^{-\text{1}}$
Dominant knee strength	Maximum dominant side knee strength value out of three trials	N.m	${\left(\text{M}.\text{g}.{\text{l}}_{\text{1}}\right)}^{-\text{1}}$
**Bipedal standing balance**			
RMS(CoP)_EO_	Average standing postural sway with eyes open on both legs	m	${\left({\text{l}}_{\text{1}}\right)}^{-\text{1}}$
RMS(CoP)_EC_	Average standing postural sway with eyes closed on both legs	m	${\left({\text{l}}_{\text{1}}\right)}^{-\text{1}}$
RMS(CoP)_EC/EO_	Romberg ratio of the standing postural sway on both legs	–	–
Path(CoP)_EC_	Amount of movement in the CoP with eyes open on both legs	m	${\left({\text{l}}_{\text{1}}\right)}^{-\text{1}}$
Path(CoP)_EO_	Amount of movement in the CoP with eyes closed on both legs	m	${\left({\text{l}}_{\text{1}}\right)}^{-\text{1}}$
Path(CoP)_EC/EO_	Romberg ratio of the amount of movement in the CoP when standing on both legs	–	–
**Unipedal standing balance**			
RMS(CoP)_Dominant_	Average standing postural sway with eyes open on the dominant leg	m	${\left({\text{l}}_{\text{1}}\right)}^{-\text{1}}$
RMS(CoP)_NonDominant_	Average standing postural sway with eyes open on the non-dominant leg	m	${\left({\text{l}}_{\text{1}}\right)}^{-\text{1}}$
Duration(balance)_Dominant_	Duration of balance on the dominant leg	sec	${\left(\sqrt{{\text{l}}_{\text{1}}/\text{g}}\right)}^{-\text{1}}$
Duration(balance)_NonDominant_	Duration of balance on the non-dominant leg	sec	${\left(\sqrt{{\text{l}}_{\text{1}}/\text{g}}\right)}^{-\text{1}}$
**Gait parameters**			
Gait speed	Distance traveled per time unit	m.sec^-1^	${\left({\text{l}}_{\text{1}}.\sqrt{{\text{l}}_{\text{1}}/\text{g}}\right)}^{-\text{1}}$
Cadence	Number of steps per time unit	sec^-1^	$\sqrt{{\text{l}}_{\text{1}}/\text{g}}$
Stride length	Sagittal distance between successive heel strikes of same foot	m	${\left({\text{l}}_{\text{1}}\right)}^{-\text{1}}$
Step width	Lateral distance between successive heel strikes of two feet	m	${\left({\text{l}}_{\text{1}}\right)}^{-\text{1}}$
Gait stability ratio	Cadence divided by gait speed	m^-1^	${\text{l}}_{\text{1}}$
Single support	One foot in touch with the ground	%	–
Double support	Both feet in touch with the ground	%	–
**Dynamic gait balance**			
StepLength_right_	Distance between two consecutive steps on the right side	m	${\left({\text{l}}_{\text{2}}\right)}^{-\text{1}}$
StepLength_left_	Distance between two consecutive steps on the left side	m	${\left({\text{l}}_{\text{2}}\right)}^{-\text{1}}$
DSM_right_	Shortest distance from the xCoM to the BoS during the gait cycle for the right leg	m	${\left({\text{l}}_{\text{2}}\right)}^{-\text{1}}$
DSM_left_	Shortest distance from the xCoM to the BoS during the gait cycle for the left leg	m	${\left({\text{l}}_{\text{2}}\right)}^{-\text{1}}$

*Abbreviations: M: Body mass (units: Kg);* $l_{\text{1}}$*: Leg length (units: m); g: gravity (units: m.s*^*-2*^*);* $l_{\text{2}}$*: Height (units: m)*.

**Fig 3:**
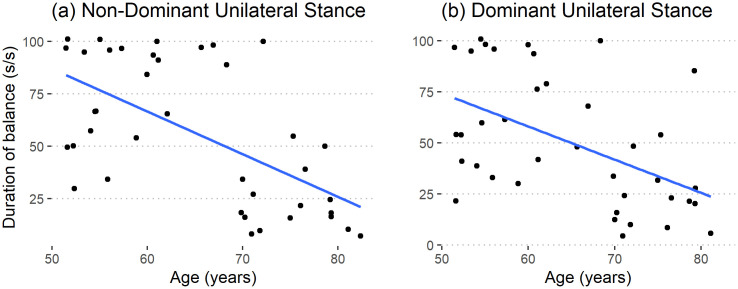
Unipedal standing duration for the (a) non-dominant (R^2^ = 0.38, p < 0.001) and (b) dominant sides (R^2^ = 0.27, p = 0.004).
